# Normalization of TAM post-receptor signaling reveals a cell invasive signature for Axl tyrosine kinase

**DOI:** 10.1186/s12964-016-0142-1

**Published:** 2016-09-06

**Authors:** Stanley G. Kimani, Sushil Kumar, Viralkumar Davra, Yun-Juan Chang, Canan Kasikara, Ke Geng, Wen-I Tsou, Shenyan Wang, Mainul Hoque, Andrej Boháč, Anita Lewis-Antes, Mariana S. De Lorenzo, Sergei V. Kotenko, Raymond B. Birge

**Affiliations:** 1 Rutgers, Department of Microbiology, Biochemistry and Molecular Genetics, Cancer Center, Rutgers- New Jersey Medical School, 205 South Orange Ave, Newark, NJ 07103 USA; 2 Rutgers, Biomedical and Health Sciences, OIT/High Performance and Research Computing, 185 South Orange Ave, Newark, NJ 07103 USA; 3 Rutgers, Department of Microbiology, Biochemistry and Molecular Genetics, Genomics Research Program, Rutgers- New Jersey Medical School, 185 South Orange Ave, Newark, NJ 07103 USA; 4 Department of Organic Chemistry, Comenius University in Bratislava, Faculty of Natural Sciences, Mlynská dolina, Ilkovičova 6, 842 15 Bratislava, Slovakia; 5 Biomagi, Ltd, Mamateyova 26, 851 04 Bratislava, Slovakia

**Keywords:** TAM RTKs, Signaling, Invasion, Metastasis

## Abstract

**Background:**

Tyro3, Axl, and Mertk (TAMs) are a family of three conserved receptor tyrosine kinases that have pleiotropic roles in innate immunity and homeostasis and when overexpressed in cancer cells can drive tumorigenesis.

**Methods:**

In the present study, we engineered EGFR/TAM chimeric receptors (EGFR/Tyro3, EGFR/Axl, and EGF/Mertk) with the goals to interrogate post-receptor functions of TAMs, and query whether TAMs have unique or overlapping post-receptor activation profiles. Stable expression of EGFR/TAMs in EGFR-deficient CHO cells afforded robust EGF inducible TAM receptor phosphorylation and activation of downstream signaling.

**Results:**

Using a series of unbiased screening approaches, that include kinome-view analysis, phosphor-arrays, RNAseq/GSEA analysis, as well as cell biological and in vivo readouts, we provide evidence that each TAM has unique post-receptor signaling platforms and identify an intrinsic role for Axl that impinges on cell motility and invasion compared to Tyro3 and Mertk.

**Conclusion:**

These studies demonstrate that TAM show unique post-receptor signatures that impinge on distinct gene expression profiles and tumorigenic outcomes.

**Electronic supplementary material:**

The online version of this article (doi:10.1186/s12964-016-0142-1) contains supplementary material, which is available to authorized users.

## Background

Tyro3, Axl, and Mertk (abbreviated TAMs) are a family of three homologous type I receptor tyrosine kinases that have important roles in innate immunity and in the oncogenic transformation of tumor cells [1–7]. Structurally, TAMs share a conserved extracellular domain comprised of two tandem immunoglobulin-like (Ig) domains and two tandem fibronectin type III (Fn-III) domains, followed by a single trans-membrane spanning region and an intracellular tyrosine kinase domain [2, 8–10]. The best-characterized ligands for TAMs are Growth Arrest Specific Factor-6 (Gas6) and Protein S (Pros1) that bind to the extracellular Ig domains of TAMs and induce dimerization, tyrosine phosphorylation, and post-receptor activation of downstream signaling pathways [11–13]. Both Gas6 and Pros1 interact with externalized phosphatidylserine (PS) on apoptotic cells [14–17] and enveloped viruses [18–23] via their γ-carboxylated Gla domain, thereby enabling TAMs to serve indirectly as PS receptors for the clearance of apoptotic cells and for viral entry.

While all three TAMs promote apoptotic cell clearance and viral entry via the interactions with Gas6 and Pros1, TAMs have different specificities and affinities towards their ligands, exhibit different tissue expression patterns, and their promoters are regulated by distinct extracellular stimuli [24, 25]. Axl, which is more prominently expressed on bone-marrow derived dendritic cells (BMDCs), is up-regulated under pro-inflammatory conditions, and has high affinity for Gas6 (Kd in nM range) but undetectable affinity for Pros1 [16, 26, 27]. On the other hand, Mertk is more prominently expressed on M2 macrophages, is induced under tolerogenic and anti-inflammatory conditions and down-regulated by LPS, binds both Gas6 and Pros1 with lower affinities (Kd in uM range) [26, 28]. Tyro 3, which is the most widely expressed and abundant member of the TAM family, is highly expressed in the nervous system, also binds Gas6 and Pros1, although there appears to be preferential specificity for Pros1 [25, 26, 29]. TAMs also display different requirements for PS, whereby Mertk and Tyro3 can be hyper-activated by their ligands in the presence of PS-positive apoptotic cells or liposomes [16, 24, 25, 30]. However, despite such broad and dynamic expression patterns that includes immune cell subsets of both myeloid and lymphoid origin, vascular endothelial cells, epithelial cells, cells of the reproductive tissues, neuronal cells, as well as mesenchymal and neuronal stem cells [2, 3, 31], TAMs are nonessential for embryogenesis, and single, double or triple knockouts are viable without visible developmental and perinatal defects. However, during post-pubescent aging, TAM knockouts display chronic inflammation and autoimmune type disorders reminiscent of systemic lupus erythematosus (SLE) [31–34]. Collectively, in adults, TAMs have specialized homeostatic functions that control the tolerogenic clearance of apoptotic cells and the resolution and maintenance of inflammation [24].

Adding complexity, TAMs also participate in a variety of heterotypic interactions with specific cytokine receptors, integrins, and cell adhesion molecules to profoundly influence receptor versatility. For example, Axl has been shown to participate in a heterotypic interaction with the Interferon type I receptor (IFNAR1) to activate Stat1 and negatively regulate inflammatory cytokine signaling via the expression of SOCS1 and SOCS3 [4]. Mertk, on the other hand, functionally interacts with αvβ5 integrin, to induce phagocytic uptake of apoptotic cells and rod outer segments [35]. The interface between TAMs, their ligands, and co-receptors diversify the repertoire of signaling of this RTK family.

In addition to their complex regulation under physiological conditions, all three TAMs are also strongly implicated in human cancers, whereby aberrantly elevated expression and signaling is often associated with cancer progression, metastasis, and resistance to targeted therapies [1–3, 36–38]. Indeed, many cancers, including cancers of the breast, colon, glioblastomas, kidneys, leukemia’s, liver, lung, melanomas, multiple myelomas, osteosarcomas, ovaries, prostate, stomach, thyroid and uterine endometrium display dys-regulated expression of one or more TAM receptor tyrosine kinases [1–3]. From a therapeutic standpoint, TAMs are interesting receptor targets in cancer biology since their expression on innate myeloid cells in the cancer microenvironment leads to immune subversion, while their expression on tumor cells can drive oncogenic transformation and survival.

Here we query a fundamental question in TAM biology as to whether TAM specificity is achieved intrinsically at the level of post-receptor activation of downstream signaling. To address this issue, we developed reporter cell lines to study TAM post-receptor signaling in a normalized ligand-inducible system that eliminates differences in ligand-specific TAM activation. While previous studies have utilized various chimeric receptors, such as EGFR/Tyro3 [13, 39], EGFR/Axl [40–42], EGFR/Mertk [43], FMS/Mertk [44] and CD/Mertk [35, 45] to map early post-receptor signals and demonstrate the fidelity of TAM chimeric approaches, here we expressed EGFR-TAMs in the EGFR negative CHO cells to systematically query whether, upon ligand engagement, TAMs have overlapping or unique post-receptor signaling signatures. By interrogating post-receptor pathways using kinome-view, phosphor-arrays, RNA-Seq and subsequent biological validation, we show that TAMs activate unique intrinsic post-receptor signaling pathways devoid of their differential reliance on endogenous ligands Gas6 and Pros1. Moreover, these studies provide a rationale for the metastatic activity of Axl, whereby EGFR-Axl expressing cells preferentially activate a tumorigenic signature that promotes cytoskeletal reorganization, motility, invasion, and in vivo, Axl knock-out in the triple negative breast cancer cell line 4 T1 showed impaired tumor growth and subsequent decreased frequency of metastasis to the lung.

## Methods

### Antibodies and reagents

Antibodies used were as follows: anti-hEGFR (Santa Cruz, Sc-120), PE-Mouse anti- hEGFR (BD Biosciences, 555997), anti-hTyro3 (Cell Signaling, D38C6), anti- phosphor-hTyro3 (Aviva Systems Biology, OAAF00456), anti-hAxl (Cell Signaling, C89E7), anti-phospho-Axl (Cell Signaling, D12B2), anti-hMer (Cell Signaling, D21F11), anti-phospho-Mer (FabGennix, PMKT-140AP), anti-phospho-STAT1 (BD Bioscience, 612233), anti-phospho-Akt (Ser473) (Cell Signaling, 193H12), anti-phospho-Akt (Thr308) (Cell Signaling, D25E6), anti-phospho-Erk1/2 (Cell Signaling, 20G11), anti-β-Actinin (Cell Signaling, MAB374), and anti-phosphotyrosine pY99 (Millipore, 05–321), anti-hGas6 (R&D Systems, AF986), Human EGF (Invitrogen, PHG0311). The secondary antibodies used for immunoblot analysis were horseradish peroxidase–conjugated Affinipure Goat anti-mouse (Jackson ImmunoResearch, 115-035-166) and anti-rabbit (Jackson ImmunoResearch, 111-035-144).

### Generation of chimeric receptor constructs and reporter cell lines

The cDNA fragments encoding TAMs transmembrane and intracellular domains were amplified by nested PCR from human testis cDNA library using sequence specific primers flanked with restriction enzyme sites at the ends (see Additional file 1). PCR-generated DNA fragments were digested with EcoR1 and Nhe1 and cloned into corresponding sites of pEF2-FLCRF2-12/IFN-γR1 plasmid [46] to replace the intracellular domain of IFN-γR1. The cDNA fragment encoding EGFR extracellular domain was amplified by PCR from human EGFR containing plasmid (Additional file 1). PCR-generated DNA fragments were digested with Kpn1 and Nhe1 and cloned into corresponding sites of pEF2- FLCRF2-12 /TAMs plasmid to replace the extracellular domain of FLCRF2-12. The cloning generated pEF2-EGFR/hTyro3, pEF2-EGFR/hAxl and pEF2-EGFR/hMertk plasmids, which contain extracellular domains of human EGFR and human TAM transmembrane and intracellular domains. The constructs were transfected into 16–9 CHO cells, a cell line that showed undetectable expression of any of the three TAMs. Stable single cell derived clonal populations were generated following G418 selection and chimeric receptors expression was determined.

### Detection of activation of chimeric TAM Receptors

Serum starved, stable EGFR/TAM CHO cells were stimulated with 100 ng/ml hEGF for 5 min and whole cell lysates were prepared in HNTG buffer (20 mM HEPES, pH 7.5, 150 mM NaCl, 10 % glycerol, 1 % Triton X-100, 1 mM PMSF, 1 mM Na_3_VO_4_, 10 mM Na_2_MoO_4_, 1 mM EDTA, 10 mM NaF and 20 μg/mL aprotinin) and processed by immunoblotting. EGFR/TAM activation was measured by pTyro3, pAxl or pMertk signal intensities and normalized to respective β-Actin protein loading controls.

### Detection of surface EGFR expression by flow cytometry

Parental CHO 16–9 cells and stable cell lines expressing chimeric EGFR/hTYRO3, EGFR/hAXL and EGFR/hMERTK were seeded in 10-cm dishes and allowed to attain 80 % confluency. The cells were washed three times in PBS without calcium and magnesium, incubated with Accutase® (Innovative Cell Technologies) and collected in FACS stain buffer (PBS with 1 % FBS). The cells were washed three times in FACS stain buffer, counted and 1 × 10^7^ cells/mL resuspended in 100 μl FACS stain buffer. The resuspended cells were incubated with PE mouse Anti-Human EGF receptor antibody for 30 min on ice according to the manufacture’s protocol. The cells were then rinsed three times in cold PBS and the cell pellet resuspended in 0.5-mL of FACS Stain Buffer. The stained cell samples were analyzed by flow cytometry (BD FACSCalibur™) and data analyzed using BD CellQuest Pro software.

### Kinome-view profiling

Serum starved, stable EGFR/TAM CHO cells were stimulated with hEGF for 0, 5 and 30 min. The cells were then lysed in urea buffer (9 M urea, 20 mM Hepes (pH 8.0), 1 mM sodium orthovanadate, 2.5 mM sodium pyrophosphate, and 1 mM β-glycerophosphate). 14 μg of total protein was run in each lane for immunoblotting. The blots were probed with motif antibodies designed to provide a Kinome-wide view of cellular phosphorylation. Immunoblots were developed using a LI-COR Odyssey NIR (near infrared) imaging system.

### Human phospho-kinase array

Human Phospho-kinase arrays were performed according to manufacturer’s instructions (R&D Systems). Briefly, serum starved, stable EGFR/TAM CHO cells were stimulated with hEGF for 30 min, lysed and 300 μg of total protein incubated overnight at 4 °C. Membranes were subsequently washed and subjected to the antibody array. The membranes were washed again and then exposed to chemiluminescent reagent and quantification of pixels was performed by densitometry using Alpha View software.

### RNA seq, heatmap plot and gene enrichment analysis (GSEA)

Serum starved, stable EGFR/TAM CHO cells were stimulated with hEGF for 6 or 24 h. Total cellular RNA was extracted and analyzed for integrity and samples with RNA integrity number (RIN) >9.0 were used for subsequent processing. Total RNA was subjected to two rounds of poly(A) selection using oligo-d(T)25 magnetic beads. A single-read (strand specific) cDNA library was prepared following the Illumina TrueSeq small RNA protocol for strand-specific RNA-seq with minor modifications [47]. Briefly, poly(A) + RNA was fragmented in an alkaline buffer (NaHCO_3_ at pH 9.3) for 2 min at 94 °C followed by dephosphorylation with recombinant shrimp alkaline phosphatase and phosphorylation with T4 polynucleotide kinase. After addition of 3′ adapter (5′ adenylated) and 5′ adapter using truncated T4 RNA ligase II and T4 RNA ligase I, respectively, RNA was reverse-transcribed using 3′ adapter-specific primer. cDNA was then amplified by PCR for 15 cycles with a universal forward primer and a reverse primer with bar code. The cDNA libraries were purified using AmpureXP beads and quantified on an Agilent Bioanalyzer (Additional file 1). Sequencing was done on NextSeq 500 Illumina with 1x75 configuration. Raw reads were quality trimmed using Trimmomatic-0.33 with leading and trailing Q score 25, minimum length 25 bp, and adaptors were removed. The cleaned reads were mapped to Cricetulus griseus genome, then aligned reads were counted, both using CLC Genomics Workbench 8.5.1 with CLC Genomics Server 7.0. The reference genome sequence and annotation files were downloaded from NCBI (ref_CriGri_1.0_chrUn.fa; cgr_ref_CriGri_1.0_chrMT.fa; ref_CriGri_1.0_top_level.gff3). The bioconductor package edgeR_3.8.6 with limma_3.22.7 was used to perform the differential gene expression analysis, under R environment, R version 3.1.2. Heatmap was plotted using the log2 transformed CPM expression values within R heatmap_2 function. Gene sets were created specifically for Chinese hamster genome using either genes from KEGG pathways, or gene selected and annotated based on knowledge. Genes were pre-ranked using an in-house script, then the GSEA Preranked analysis was conducted [48].

### RT-qPCR

cDNA of Cricetulus griseus mRNA was made using high capacity cDNA reverse transcription kit using random primers (Applied Biosystems, Warrington, UK). Real-time RT-PCR was performed on a Rotorgene RG-2000 (Corbett Research, Mortlake NSW, Australia) by using the Access RT-PCR system (Promega) according to the manufacturer’s instructions (Additional file 1).

### Adhesion, migration and invasion assays

Real-time cell adhesion, migration and invasion were determined using Xcelligence system, as previously described [49]. Briefly, respective cells were serum starved overnight in medium containing 0.5 % FBS, counted and resuspended in serum-free medium (SFM). In the lower chamber of the CIM plate, respective media containing 10 % FBS was added as chemoattractant. In the top chamber, 50 μl of SFM was added to all wells for equilibration step. 100 μl of each cell line suspended in SFM (40,000 cells/100 μl) ± EGF were added in triplicates in upper chamber wells. Changes in the cell index (CI) depicting cellular migration, were assessed every 10 min for 24 h and were shown as a change in cell index versus time. For cell invasion assays, an additional 10 % matrigel plug was added in the upper chamber.

### Axl knockout, cell proliferation and in vivo mice studies

Axl knockout cells were prepared by transfecting 4 T1-luc2-GFP mouse breast cancer cells with two clones of All-in-one guide RNA (Additional file 1) and flow sorted for RFP expression. Single cell clones were grown and screened by immunoblotting and surveyor assay. For cell proliferation MTT assay, 4 × 10^3^ wild-type (WT) or Axl KO 4 T1-luc2-GFP cells per well were plated in a 48 well plate and incubated for 24–96 h in pentaplicate. After the respective time points, MTT (0.5 mg/mL) was added to each well and incubated for 4 h at 37 °C and the formazan crystals were then dissolved in 250 μl DMSO for 30 min at room temperature in the dark. The absorbance was measured at 490 nm and the cell proliferation expressed as optical density. For in vivo mice studies, 5 × 10^4^ WT or Axl KO 4 T1-luc2-GFP cells were resuspended in matrigel and injected in mammary fat pad of BALB/C mice (8 mice/group). Tumor volume and body weights were measured twice and once a week respectively. 5 weeks after the injection, mice were euthanized and primary tumor and lungs were harvested. Metastatic index was calculated by counting metastatic nodules in the lungs. The mouse experiments were done in accordance with guidelines and under approval from IACUC.

### Data analysis

Statistical analysis was performed using GraphPad Prism. Descriptive statistics for quantitative variables were summarized using mean ± SD or mean ± SEM. Differences between groups were tested and differences with a P value of <0.05 were considered statistically significant.

## Results

### Development of chimeric EGFR-TAM reporter cell lines in EGFR-negative hamster CHO cells

**Fig. 1:**
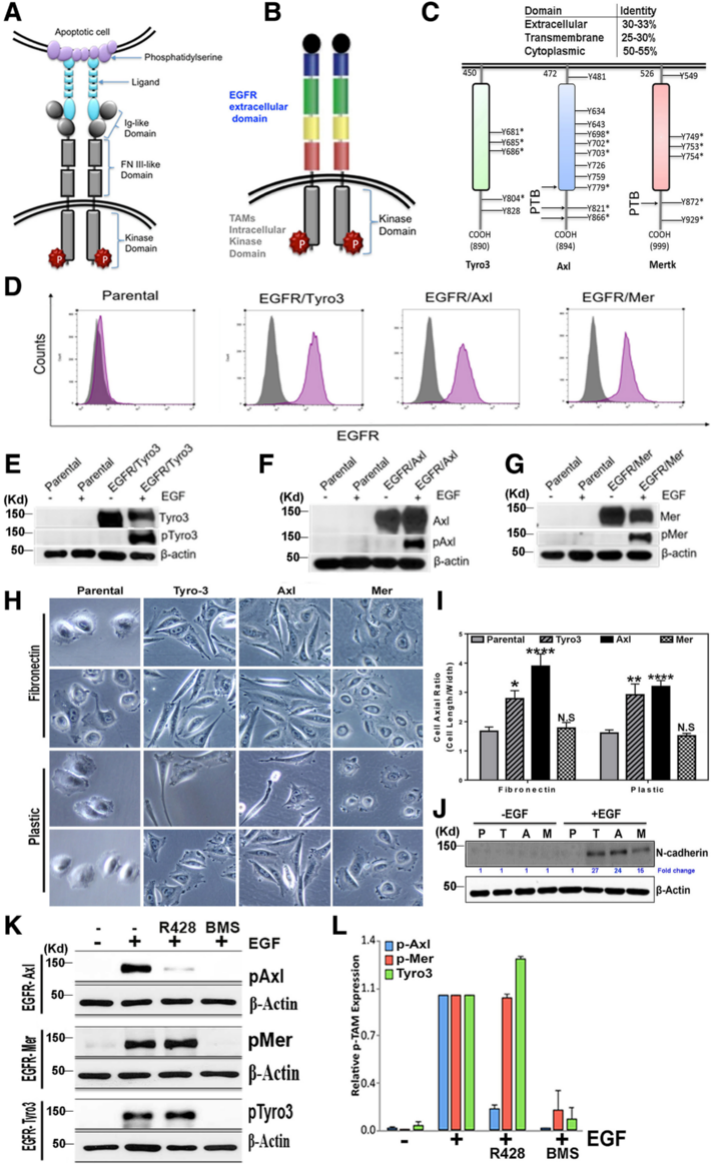
Characterization of EGFR/TAM chimeric receptors. **a** Schematic representation of wild-type TAM receptors. The ligands, (Gas6 and Pros1) serve as bridging molecules to link TAMs on phagocytic cell to externalized PS on apoptotic cells. **b** Schematic representation of EGFR/TAM chimeric receptors created by fusing the extracellular domains human EGFR with the trans-membrane and intracellular domains of each TAM receptor. **c** The percentage identity between the different domains of TAMs and the different tyrosine-based motifs on TAMs intracellular kinase domain that can be phosphorylated (the *asterisks* indicate the autophosphorylation sites). **d** EGFR expression on stable EGFR/TAM cell lines as analyzed by flow cytometry. **e**-**g** Immunoblots analysis of stable EGFR/Tyro3 (**e**), EGFR/Axl (**f**) and EGFR/Mertk (**g**) CHO cell lines characterizing receptor expression, and EGF-inducible dimerization and activation of functional proteins verified using pTyro3 (**e**), pAxl (**f**), and pMertk (**g**) antibodies. **h** Representative bright-field micrographs of parental and EGFR/TAM CHO cells seeded on fibronectin-coated (*upper panels*) or uncoated (*lower panels*) plastic surface. The Inset shows a representative enlarged single cell. **i** The cell axial ratio (cell length/width) quantification data of parental and EGFR/TAM CHO cells seeded on fibronectin-coated or plastic surface. Differences between groups were tested by two-way ANOVA and Tukey’s multiple comparisons test, *P < 0.05, **P < 0.01, ****P < 0.0001. **j** Immunoblot analysis of N-Cadherin induction by TAMs. **k** Immunoblot analysis showing effects of 200nM R428 and 200nM BMS777607 on TAMs activation. **l** Densitometry analysis of the immunoblots showing the percentages of inhibition compared to EGF treatment only. Mean values ± SD are shown (*n* = 3)

Previously, we developed TAM-IFNγR1 chimeric reporter cell lines to interrogate the activation of TAM receptors by their endogenous ligands [25]. These studies showed that TAMs have distinct ligand-inducible activation patterns by Gas6 and Pros1, as well as differential requirement for hyper-activation in the presence of PS-positive apoptotic cells or PS liposomes. Here, we have taken a conceptually similar approach and engineered a new set of chimeric EGFR-TAM gene products by fusing the extracellular domain of human EGFR in frame with the trans-membrane and intracellular domains of each human TAM receptor (Fig. 1 a, b). TAMs share ~50–55 % identity in their kinase domains, although there is less conservation in their trans-membrane regions (20–25 %) and cytoplasmic tails, the latter of which contains unique autophosphorylation sites for SH2/PTB domain binding [3] (Fig. 1c). By expressing EGFR/TAM receptors in EGFR and TAM negative CHO cells, post-receptor signaling is exquisitely dependent on EGF stimulation, allowing for normalization of TAM post-receptor signaling not possible using native receptors, given native receptors respond differentially to endogenous ligands.

In Fig. 1d, native CHO cells (parental) were transfected with EGFR/Tyro3, EGFR/Axl, or EGFR/Mertk expression plasmids, and after neomycin selection, pooled stable lines were FACS-sorted with anti-EGFR antibodies that bind native surface EGFR [50]. Subsequently, geometric mean intensity gating was used on single cell clones to ensure equal surface expression of chimeric receptors (Fig. 1d). After serum starvation for 16 h, parental or EGFR/TAM CHO cells were stimulated for 5 min with 100 ng/ml EGF, and detergent lysates prepared and immunoblotted with native anti-Tyro3 or anti-phosphoTyro3 (Fig. 1e), native anti-Axl or anti-phosphoAxl (Fig. 1f), or native anti-Mertk or anti-phosphoMertk antibodies (Fig. 1g). Under these conditions, EGFR/TAMs maintain minimal, if any, level of receptor activation in the absence of ligand (EGF), and demonstrate robust and immediate activation following stimulation with EGF (compare upper and lower panels in Fig. 1, e-g).

Despite the fact that each EGFR/TAM was expressed at an equal level by sorted populations as measured by geometric mean-intensities, each TAM displayed different morphologies on plastic and fibronectin (FN) (Fig. 1h, i). In this capacity, EGFR/Axl and EGFR/Tyro3 preferentially showed fusiform-like spindle shaped cells on both plastic and FN, although EGFR/Axl cells enhanced this phenotype when cultured on FN. In contrast, EGFR/Mertk showed a fusiform-independent morphology, similar to parental cells, even after TAM activation on FN (Fig. 1, h-i). Moreover, and consistent with the fusiform morphology, while all three chimeric lines collectively induced the EMT marker, N-cadherin, this effect was most prominent in the EGFR/Axl and EGFR/Tyro3 cell lines (Fig. 1j).

Furthermore, since TAMs have been intensely interrogated as targets of small molecule tyrosine kinase inhibitors, we evaluated whether EGFR/TAM lines could be used as a screening tool for TAM antagonists. Indeed, as has been reported for native TAMs [1], EGFR/TAM chimeric receptors maintain selectivity in their responsiveness towards tyrosine kinase inhibitors. For example, the Axl-specific inhibitor R428 (BGB324) [51] showed selectivity for Axl (Fig. 1k, l), whereby only EGFR/Axl cells were inhibited (minimal inhibition of other lines at up to 1 μM inhibitor concentration). In contrast, when cells were pretreated with the pan-TAM inhibitor BMS-777607 [52], all three EGFR/TAMs were equally inhibited in this assay (Fig. 1k, l). These data suggest that EGFR/TAM lines have utility in drug screening, and by inference, each TAM kinase domain has distinct elements for post-receptor activation and substrate-level phosphorylation, even when the tyrosine kinase activation is normalized with respect to ligand-induced dimerization, kinetics of phosphorylation of the activation loop, and strength of signaling.

### Prominent role of Axl in regulation of ligand-induced cytoskeletal changes and spontaneous metastasis to lung

**Fig. 2:**
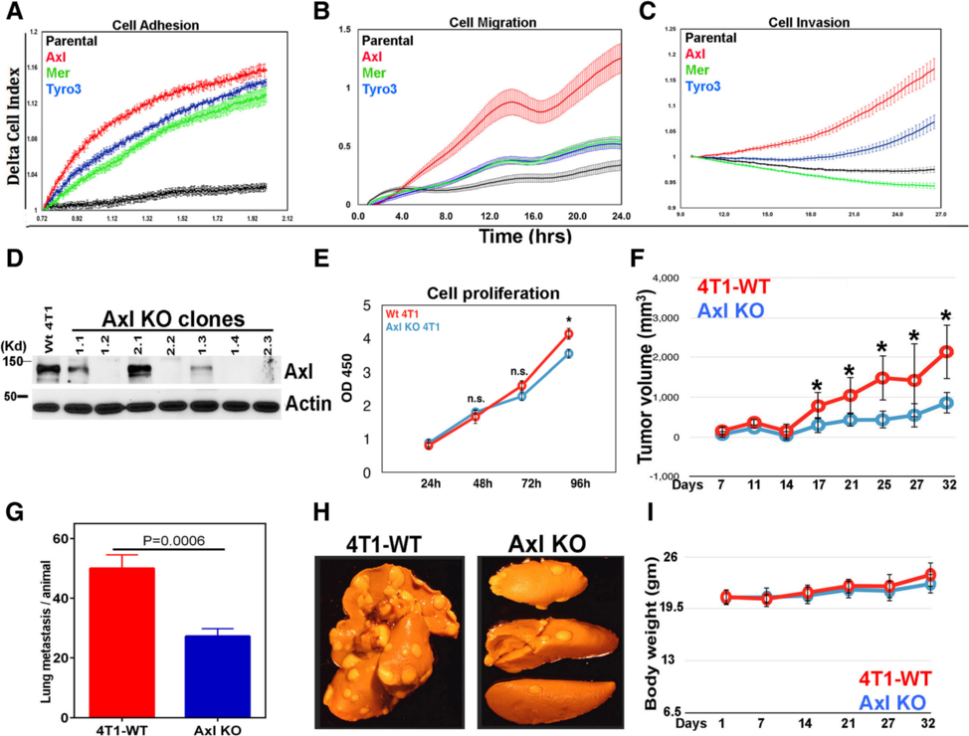
Effects of Tyro3, Axl and Mertk on Cell adhesion, migration and invasion. **a**-**c** Parental and EGFR/TAM CHO cells adhesion (**a**), migration (**b**) and invasion (**c**), in response to EGF stimulation analyzed by xCELLigence system. **d** Immunoblot analysis of Axl expression showing CRISPR/Cas9 efficiently disrupts Axl in 4 T1 cell clones 1.2, 1.4, 2.2 and 2.3. 4 T1 Axl knockout clone 2.2 was used in subsequent experiments. **e** The effect of Axl knockout on cell growth compared to 4 T1 WT as determined by assessing cell proliferation using an MTT assay for upto 96 h. Mean values ± SD are shown (*n* = 5). **f** BALB/C mice were injected in mammary fat pad with 5 × 10^4^ 4 T1 WT or 4 T1 Axl KO clone 2.2 cells, 8 mice per group. On day 7 after cancer cell implantation, tumor volume measurements began and were performed every 3 days. Mean ± SD is shown, *P < 0.05 by Student’s *t*-test. **g** Quantification of microscopic nodules in the lungs of each group and data presented as the mean ± SE. Statistical analysis was performed using Student’s t- test. **h** Representative lung samples showing metastatic nodules in 4 T1 WT vs 4 T1 Axl KO groups. **i** Total body weights were not significantly different between the 4 T1 WT and 4 T1 Axl KO groups

To translate the contribution of each EGFR/TAM to specific cell biological outcomes, we examined effect of EGF stimulation (i.e. TAM activation) on cell adhesion (Fig. 2a), cell migration (Fig. 2b) and cell invasion (Fig. 2c) using real-time Xcelligence technology (see methods). All three EGFR/TAM lines showed enhanced adhesion on a FN-coated surface, suggesting each TAM impinged on cytoskeletal reorganization required for early cell adhesion events. In contrast, when EGFR/TAM CHO cells were monitored for motility and invasion, each TAM showed differential responsivity, whereby EGFR/Axl showed greatest effects on chemotactic (migration) activity and invasive activity through a 10 % matrigel membrane. Notably, CHO cells are not considered invasive (i.e. capable of degrading a 10 % Matrigel plug). These data suggest that Axl (and to a lesser extent Tyro3, but not Mertk) activates an intrinsic post-receptor events associated with invasion, an important hallmark of metastatic dispersion of primary tumor cells (Fig. 2b, c).

To extend observations from EGFR/TAM chimeric receptors, and to validate effects of Axl on motility and invasion on native full-length receptors, we utilized CRISPR/Cas9 technology to specifically knockout Axl in the murine 4 T1 cells (an aggressive triple negative breast cancer cell line that expresses TAMs, Axl being more abundant than Tyro3 and Mertk [53]) (Fig. 2d). In vitro cell proliferation analysis using MTT assay revealed there were no significant differences in growth characteristics between 4T1 WT and Axl KO at 24, 48 and 72 h and only a small but significant decrease (<15 %) at 96 h (Fig. 2e). However, when 5 × 10^4^ cells (clone 2.2) were transplanted into the mammary fat pad of an immune-competent BALB/C host, AXL KO showed impaired tumor growth (50 % less tumor volume, Fig. 2f), which correlated with less metastasis (50 % less metastatic burden in the lungs, Fig. 2g, h). Mice injected with Axl KO and 4T1 WT cells did not differ in average body weights (Fig. 2i). Collectively, these data suggest that Axl intrinsically activates an invasive/metastatic pathway in epithelial cells, consistent with previous reports that demonstrated the essential role of Axl in EMT transition in breast cancer metastasis [54] and also in EGFR-targeted drug-resistant tumors [55].

### Differential regulation of post-receptor signaling by TAM receptors

**Fig. 3:**
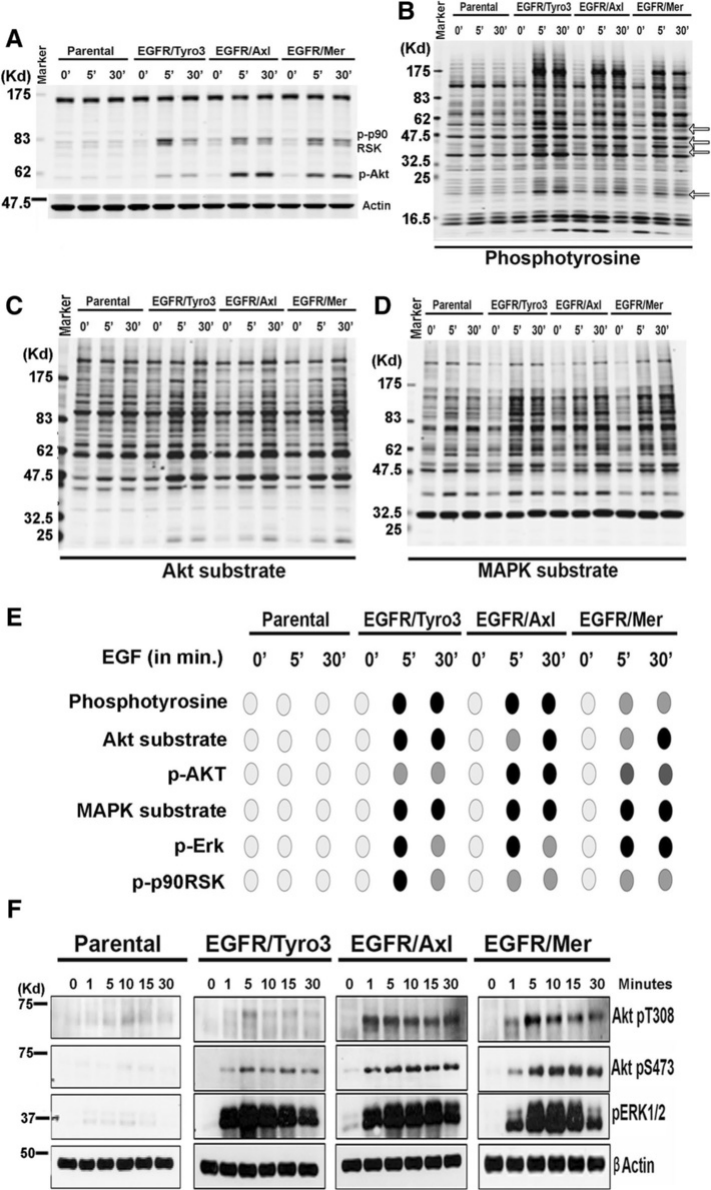
Phosphoproteomic profiling reveals specific Tyro3, Axl or Mertk dependent changes in protein phosphorylation. Parental and EGFR/TAM CHO cells were stimulated with EGF for the indicated time points. **a** Representative PathScan® Multiplex immunoblot analysis of the phosphorylation patterns of p90RSK and Akt upon TAMs activation for 0, 5 or 30 min. **b** Immunoblotting analysis of total protein tyrosine phosphorylation using anti-p-Tyr (pY1000) antibody. **c** Immunoblotting analysis of Akt substrate phosphorylation using RXX(s/t), RXRXX(s/t) motif antibody. **d** Immunoblotting analysis of MAPK substrate phosphorylation using (PXsP) motif antibody. **e** Summary of the KinomeView profiling showing the temporal phosphorylation profiles induced by respective TAM activation. **f** Immunoblotting analysis of time dependent changes in p-Akt (pT308), p-Akt (pS473) and p-Erk1/2 (pT202/pY204) following 0, 1, 5 10, 15 or 30 min EGF stimulation, with β-actin as the loading control

To better understand the mechanisms by which Axl impinges on invasive and metastatic itineraries, and identify molecular targets involved in post-TAM signaling, we analyzed detergent lysates from native and EGFR/TAM stimulated cells with known TAM downstream substrates, pp90RSK and pAkt, (Fig. 3a), a generalized pTyr antibody (pY1000) (Fig. 3b), as well as a series of Phospho-Motif antibodies that include AKT substrates (RXX(s/t), RXRXX(s/t)) (Fig. 3c), MAPK substrates (PXsP) (Fig. 3d) and motif antibodies recognizing substrates for AMPK, PKA/PKC, ATM/ATR, CK, CDK/tXR), that broadly detect serine, threonine, and tyrosine phosphorylation events and assess global changes in protein phosphorylation.

As shown in Fig. 3, whereas all three TAMs induced rapid tyrosine phosphorylation of RSK, Akt, and Erk, and clear inductive phosphorylation on both general tyrosine phosphorylation and substrate level phosphorylation, they do so with differing kinetics and intensities at 5 and 30 min, indicating that both the robustness and selectivity of TAM signaling can immediately diverge following kinase activation (Fig. 3). For example, in the case for Akt, which is phosphorylated following activation of all three TAMs, Akt is most robustly phosphorylated in the EGFR/Axl stimulated cells, but only marginally phosphorylated in the EGFR/Tyro3 expressing cells. To examine the phosphorylation of Akt more systematically, which can be phosphorylated on two sites; threonine 308 (Thr308) in the activation loop by protein kinase PDK1, and serine 473 (Ser473) in the C-terminal hydrophobic motif by the mTORC2 complex [56], we observed Akt to be strongly phosphorylated at Ser473 by Axl and Mertk, but weakly by Tyro3 and sustained over the 30 min analyzed. Interestingly, whereas Axl and Mertk strongly phosphorylated Akt at Thr308 albeit with differing kinetics and intensities over the 30 min, Tyro3 failed to show robust phosphorylation of Akt at Thr308, again revealing differences in Akt phosphorylation between TAMs following post-receptor activation (Fig. 3f). Similar trends were observed in the general pTyr blots where both conserved and TAM-selective phosphorylation events were observed (Fig. 3b, see arrows), as well as when lysates from the EGFR/TAM stimulated cells were analyzed by using Akt motif antibodies, MAPK motif antibodies, and anti-PKA/C motif antibodies (Fig. 3c-d, summarized in Fig. 3e). These data indicate that TAM signaling diverges immediately following post-receptor activation.

However, in contrast to the clear up-regulation of phosphorylation events associated with TAM activation, in contrast, we found no evidence for post-activation mediated ubiquitination using a broadly reactive anti-ubiquitin antibody to access receptor and substrate ubiquitination (data not shown), despite earlier reports that native TAMs (Axl) recruits the E3 ligase Cbl to the intracellular domain following receptor activation [57]. This suggests that for ligand-inducible TAM ubiquitination, these post-translational events are likely governed by extracellular events associated with Gas6/Pros1 induced receptor activation and may involve additional receptor components.

### TAMs differentially affect phospho-proteomics in EGF-stimulated CHO cells

**Fig. 4:**
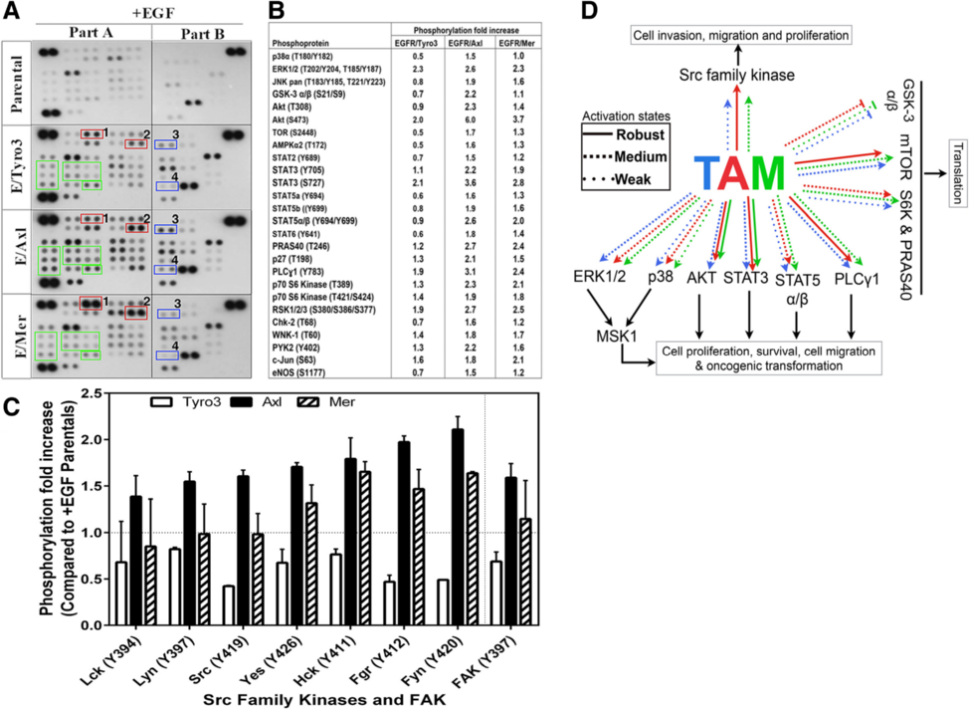
Differential phosphokinase-array profiles of activated Tyro3, Axl and Mertk. **a** Representative images of phospho-kinase array experiments from EGF stimulated parental and EGFR/TAMs total cell lysates. Activation of Tyro3, Axl and Mertk resulted in robust phosphorylation p-ERK1/2 (pT202/pY204, Box1) and p-Akt (pS473, Box2) and whereas only Axl stimulation led to robust phosphorylation of pAkt (pT308, Box3) and pSTAT3 (pS727, Box4). **b** Semi-quantitative analysis of 2 independent phospho-kinase array experiments showing the fold increase in phosphorylation in EGF stimulated over respective EGF stimulated parental spots. **c** Comparison of the fold increase in Src family kinases phosphorylation in Tyro3, Axl and Mertk, showing Axl activation results in the highest fold increase across all the Src family kinases phosphorylation (see also in **a** above, *green boxes*). **d** A summary of the signaling pathways downstream of the respective TAM activation

The aforementioned analysis showing differential phosphorylation events for each TAM as well as differences in the qualitative and quantitative detection of phosphoproteins prompted us to examine post-receptor signaling and gene expression profiling using unbiased approaches. Shown in Fig. 4 are results using a customized R&D human phospho-kinase array that detects phosphorylation of 43 human kinases and their substrates implicated in signal transduction. Samples were monitored in quadruplicate and results normalized as shown in Fig. 4a, b. All three TAM phosphorylated RSK, Akt, and Erk, albeit with different kinetics and intensity as noted above (Fig. 3e). All three TAMs also robustly activated STAT3 (S727), PLCɣ1, c-Jun and RSK1/2/3. However, phosphoarrays also revealed distinct phosphorylation events and patterns, Axl and Mer kinase domains but not Tyro3 increased the activation of JNK pan, STAT3 (Y705), STAT5b (Y699), STAT5α/β (Y694/Y699), PRAS40, p27, p70 S6 Kinase, WNK-1 and PYK2 by ≥ 1.5 fold. Interestingly, downstream phosphorylation of p38α, mTOR, AMPKα2, STAT2, STAT5a (Y694), STAT6, Chk-2, eNOS and GSK-3 α/β was only significant in Axl kinase domain activation (Fig. 4b). Notably, Axl exhibited the most robust induction in the tyrosine phosphorylation of several Src family kinases (SFKs), including Src, Fyn, Lyn, Fgr, and Lck, as well as upstream effector of Src, FAK (FAK397), when compared to Mertk and Tyro3 (Fig. 4c). This is consistent with previous reports that Axl can employ Src kinases to maximize cellular invasion in hypoxic tumors [58]. The data is summarized in Fig. 4d.

### Ligand-induced activation of Axl induce genes associated with metastatic potential

**Fig. 5:**
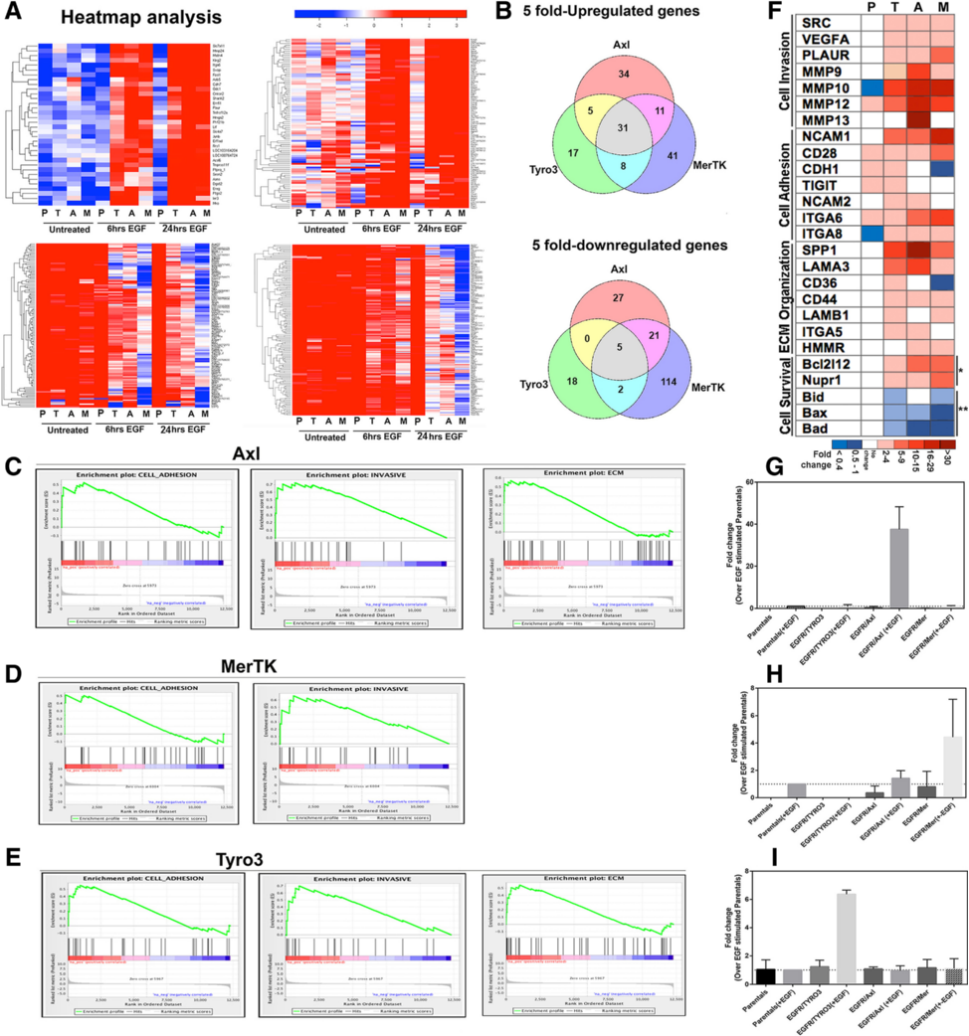
Gene-expression profiles influenced by TAMs activation. **a** Heat map showing differential expression of selected genes in Parental (P), Tyro3 (T), Axl (A) and Mertk (M) upon EGF stimulation for 6 and 24 h. RNA-Seq analysis revealed very distinct transcriptomes in EGFR/TAMs cell lines upon activation with mostly upregulated (*upper panels*) or downregulated (*lower panels*). **b** Venn diagrams showing the overlap of genes that significantly changed upon EGF stimulation for 24 h. The numbers represent a list of non-redundant genes with ≥ 5-fold, upregulation (*upper panel*) or downregulation (*lower panel*). **c**, **d**, **e** GSEA enrichment plots showing significant enrichment (ES significant at FDR < 25 %) of indicated gene signatures upregulated in Axl (**c**), Mertk (**d**), and Tyro3 (**d**) cells. **f** Heat map of the enriched genes involved cell invasion, cell adhesion, ECM organization and cell survival that displayed fold change ≥2 in gene expression in Parental (P), Tyro3 (T), Axl (A) and Mertk (M) compared to respective EGF unstimulated controls. The color indicates directionality of change in gene expression (*red* = increased, *white* = no change and *blue* = decreased). **g**-**i** RT–qPCR validation of selected genes differentially upregulated upon 24 h EGF stimulation in EGFR/Axl (**g**), EGFR/Mertk (**h**), and EGFR/Tyro3 (**i**). The genes are SPP1 in Axl (**g**), TMEM40 in Mertk (**h**), and MYH3 in Tyro3 (**i**). Expression levels are normalized to β-actin expression levels. Results shown (mean ± SD) are representative data of 3 independent experiments

Several of the phosphoproteins detected using motif antibodies (Fig. 3e) and via phosphoarray interrogation (Fig. 4a) are known to phosphorylate and activate transcription factors. To profile gene expression patterns downstream of TAMs, we performed RNAseq analysis on native and EGFR/TAM lines, following either 6 or 24 h post-EGF stimulation (Fig. 5). The RNAseq data are available in NCBI’s Sequence Read Archive (SRA) and are accessible through SRP Study accession number SRP079404. Following TAM activation, transcriptional analysis of native versus EGFR/TAM cells showed robust overall changes, with both cohorts of genes up-regulated (red) and down-regulated (blue) at both 6 and 24 h (Fig. 5a). While many of the genes showed overlapping specificity independent of the nature of the TAMs, many genes were specific to each TAM, which is illustrated in the Venn diagrams shown in Fig. 5b. Indeed, when we assessed unique versus overlapping gene sets that were up-regulated (upper panel) or down-regulated (lower panel), approximately 30 % non-redundant genes with ≥ 5-fold, up-regulation (upper panel) or down-regulation (lower panel) in response to Tyro3, Axl and Mertk activation (Fig. 5b). Pathway analysis and gene enrichment mining for pathways intrinsic to each TAM (Fig. 5c, d, e) (using both ingenuity and pathway analysis) showed each TAM enriched on regulatory pathways that impinged on cell invasion, cell adhesion, ECM organization, and cell survival (Fig. 5c, d, e). Interestingly, within these sets, Axl showed preferential up-regulation of several genes that impinge on invasion and metastasis, such as MMP10, MMP12, MMP13, SPP1, and LAMA3 (Fig. 5f). To validate genes unique to each TAM as determined by the RNAseq analysis (Fig. 5a), expression of representative genes that included SPP1 for Axl (Fig. 5g), TMEM40 for Mertk (Fig. 5h) and Myh3 for Tyro3 (Fig. 5i) was confirmed by RT-PCR. Collectively, these data suggest that TAMs are unique in post-receptor signaling, and reveal a unique invasive signature for Axl.

## Discussion

Clinically, the expression of TAM receptors in primary tumors has been associated with aggressive clinical grade, emergence of drug resistance, hallmarks of EMT, and reduced time to progression and unfavorable survival outcomes in patients [1, 2]. Moreover, TAMs are expressed on infiltrating myeloid derived cells and tumor infiltrating lymphocytes [31] and more recently TAMs have been recognized as potential inhibitory immune checkpoint receptors that suppress host tumor immune responses [57, 59, 60]. At the mechanistic level, the contribution of each TAM in tumor biology is complicated by the fact that TAMs respond with heterogeneity to their native ligands, Gas6 and Pros1, having different affinities and avidities, and different requirements for anionic lipids such as PS on apoptotic cells and liposomes. Here, we utilized chimeric TAM receptors, whereby the human EGFR extracellular domain was fused in-frame to the trans-membrane and intracellular kinase domains of each TAM receptor in order to establish a normalized cell based system to study TAM post-receptor signaling and cell biological outcomes. Interrogating this system, here we provide evidence that intracellular signaling of TAMs diverge immediately after receptor dimerization and activation, enabling TAMs to achieve selectivity in signaling via the activation of unique post-receptor signaling events. We also show that Axl-mediated post-receptor signaling triggers enhanced invasion compared to Tyro3 and Mertk, offering a partial explanation for the observed association of Axl expression with advanced-grade tumors with high metastatic potential.

Using the EGFR/TAM chimeric receptor system, whereby receptor expression and post-receptor “firing” is normalized by identical ligand-inducible events, our results provide a more detailed understanding of how TAMs achieve intrinsic specificity at the post-receptor level and conclude that each TAM has unique post-receptor functions. Evidence to support this idea comes from several observations from this study that include (i) TAMs show distinct arrangements of auto-phosphorylation sites that potentially bind SH2 and PTB-domain containing proteins (Fig. 1c), (ii) EGFR/TAMs show differential kinetics and intensities of immediate post-receptor activation profiles (i.e., pErk1/2, pAkt, and pRSK2) (Fig. 3a, e, g), (iii) EGFR/TAMs show specificity (unique) and accentuated phosphor-proteomes using unbiased phosphoarrays (R&D 43–plex) (Fig. 4), and (iv) EGFR/TAMs show non-overlapping gene expression profiles and signatures (Fig. 5). Together with previous reports showing that TAMs achieve specificity by their differential interaction with ligands, the present findings also suggest that TAM receptors have differential post-receptor activation that control different biological outcomes. Further, the notion that TAMs are functionally distinct is also supported from the pharmacological studies showing that R428 (BGB324) selectivity inhibits Axl compared to Tyro3 and Mertk [51]. This observation also points towards the utility of using EGFR/TAMs for the screening of pan-TAMs (i.e. BMS777) versus unique TAMs (i.e. R428) and validation of these small molecule therapeutics (Fig. 1k, l).

The present study also provides new insight that defines the oncogenic role of Axl in the malignant phenotypes of cancer cells. As noted above, the Axl (Gas6) axis has been reported in a multitude of human cancers, and more frequently associated with EMT and metastasis than other TAMs. Indeed, the results of unbiased proteomic and RNAseq screens not only indicated that TAMs had unique patterns of post-receptor signaling, but also identified a putative oncogenic signature for Axl that impinged on genes associated with invasion such as MMP10, MMP12, MMP13, and SPP1. EGFR/Axl activated cells also showed robust activation of Akt on Thr308 compared to Tyro3 and Mertk, as well as induced more robust and intense phosphorylation of mTOR, SFKs, and FAK, each of which is involved in metastatic cell behavior. These oncogenic signatures also translated into clear morphological and phenotypic outcomes whereby Axl activates a pro-invasion and pro-metastatic switch. When we examined phenotypic outcomes in the EGFR/TAMs with respect to cell adhesion, motility, and invasion, the EGFR/Axl-expressing cells consistently showed enhanced oncogenic parameters, and promoting invasive properties in CHO cells, a cell type not reported to have invasive properties. Finally, and consistent with previous studies using in vivo models, Axl knockout in the triple-negative 4 T1 breast cancer model, showed impaired tumor growth which correlates with decreased lung metastasis.

The observation that Axl preferentially activated several Src family kinases is consistent with previous observation showing that Src interacts with Y821 in hAxl, and in doing so, activates the Src kinase activity leading the invasive behavior of GBM cells [42]. Axl also preferentially induced tyrosine phosphorylation of FAK on Y397, a phosphorylation event that creates a docking site for Src and also linked to the activation of Src family kinases. From these data, we argue that intrinsically, of the TAMs, Axl is most likely to regulate aggressive cancer cell hallmarks associated with driver mutations, EMT, and metastasis. In contrast to Axl, Mertk induces phosphorylation of Y867 that controls crosstalk with FAK and β5 integrin to control efferocytosis (a process of engulfment of apoptotic cells) [61–63]. This may be an indication that differential activation of TAMs leads to distinct functions whereas Src family activation both in Axl and Mer leads to actin rearrangement, in Axl it is important for motility and invasion [58] whereas in Mertk it is important for PS receptor-dependent efferocytosis [64, 65], but not motility and invasion.

## Conclusion

In summary, we have found that Tyro3, Axl, and Mertk, despite a high degree of similarity in their kinase domains, exhibit specificity in post-receptor signaling, suggesting they can be uniquely exploited and targeted by anti-TAM therapeutics. These current data, combined with our previous studies showing selectivity in TAM activation by Gas6 and Pros1, provide new insight into the biology of this important family of RTKs.

## Additional file

Additional file 1: List of Primers used in this study. (PDF 180 kb)

## Abbreviations

AMPK: AMP-activated protein kinase
ATM: Ataxia telangiectasia mutated
Cbl: Casitas B-lineage lymphoma sequence
CDK: Cyclin-dependent kinase
Chk-2: Checkpoint kinase 2
ECM: Extracellular matrix
EGFR: Epidermal growth factor receptor
EMT: Epithelial-mesenchymal transition
eNOS: Endothelial nitric oxide synthase
Erk: Extracellular signal-regulated kinase
FAK: Focal adhesion kinase
Gas6: Growth Arrest Specific Factor-6
GSK-3α/β: Glycogen synthase kinase 3 α/β
hEGF: Human epidermal growth factor
IFNAR1: Interferon type I receptor
JNK: c-Jun N-terminal kinase
LAMA3: Laminin subunit alpha 3
LPS: Lipopolysaccharides
MAPK: Mitogen-activated protein kinase
MMP: Matrix metalloproteinase
mTORC2: Mammalian target of rapamycin complex 2
Myh3: Myosin heavy chain 3
PKA: cAMP-dependent protein kinase
PKC: Protein kinase C
PLCɣ: Phospholipase C gamma
PRAS40: Proline-rich Akt substrate of 40kDa
Pros1: Protein S
PYK2: Protein tyrosine kinase 2
RSK: Ribosomal s6 kinase
RTK: Receptor tyrosine kinase
SOCS: Suppressor of cytokine signaling
SPP1: Secreted phosphoprotein 1
STAT: Signal transducer and activator of transcription
TAM: Tyro3, Axl, Mertk
TMEM40: Transmembrane protein 40
WNK-1: WNK lysine deficient protein kinase 1
