# Supplementary material for: Normalization of TAM post-receptor signaling reveals a cell invasive signature for Axl tyrosine kinase
Source: Cell Commun Signal. 2016 Sep 6;14(1):19. doi: 10.1186/s12964-016-0142-1 (PMC5011882; doi:10.1186/s12964-016-0142-1)
Supplement: Additional file 1: — List of Primers used in this study. (PDF 180 kb) [file 12964_2016_142_MOESM1_ESM.pdf]

Supplementary Table T1

	List of Primers used in this study
	**Primers for nested PCR amplification of cDNA fragments encoding TAMs transmembrane and intracellular domain**
**1st round**	
Tyro3 Forward	5'-GTGGACCCTGGAGTCAGCCA-3'
Tyro3 Reverse	5'-CTGGAATTCAACAGCTACTGTGTGGCAGTAG-3'
Axl Forward	5'-GAGCCTCCCAGTACCCCTGG-3'
Axl Reverse	5'-GGTGAATTCAGGCACCATCCTCCTGCCC-3'
Mer Forward	5'-TCCCTGCACACGGTTGGGTA-3'
Mer Reverse	5'-CCTGAATTCACATCAGGACCTCTGAGCC-3'
**2nd round**	
Tyro3 Forward	5'-CTGGCTAGCAGTGGTCCTTGGTGTGCTAACG-3'
Tyro3 Reverse	5'-CTGGAATTCAACAGCTACTGTGTGGCAGTAG-3'
Axl Forward	5'-CTGGCTAGCAGTACTGCTAGGAGCAGTCGTG-3'
Axl Reverse	5'-GGTGAATTCAGGCACCATCCTCCTGCCC-3'
Mer Forward	5'-TGTGCTAGCAATCTTTGGCTGCTTTTGTGGA-3'
Mer Reverse	5'-CCTGAATTCACATCAGGACCTCTGAGCC-3'
	**Primers for PCR amplification of cDNA fragments encoding EGFR extracellular domain**
EGFR Forward	5'-CGGGTACCAGCGATGCGACCCTCCGGGA-3'
EGFR Reverse	5'-CCAGCTAGCCAGGACGGGATCTTAGGCCCATT-3'
	**Primers for PCR amplification of cDNA library**
5' Adapter	5'-GUUCAGAGUUCUACAGUCCGACGAUCNNNN
3' Adapter	5'/ 5rApp/ NNNNTGGAATTCTCGGGTGCCAAGG 3ddC/
RT Primer	5' GCCTTGGCACCCGAGAATTCCA
Forward primer	5'AATGATACGGCGACCACCGAGATCTACACGTTCAGAGTTCTACAGTCCGA
Reverse primer	Index CAAGCAGAAGACGGCATACGAGATCGTGATGTGACTGGAGTTCCTTGGCACCCGAGAATTCCA (barcode)
	**Primers for RT-PCR validation**
Tmem40 Forward	5'-AAGGACGACGAGTTCTTC-3'
Tmem40 Reverse	5'-TCGGCGTAGTAGTGGTAG-3'
Spp1 Forward	5'-CAGGAAGTTCCACATCTC-3'
Spp1 Reverse	5'-CTCCTTGGACTTCATGTG-3'
Myh3 Forward	5'-TAGTGCCATTGACATCTTG-3'
Myh3 Reverse	5'-TGCTTCTGCTTGAACTTC-3'
Actb Forward	5'-GCATCCACGAAACTACAT-3'
Actb Reverse	5'-CACTGTGTTGGCATAGAG-3'
